# A critical perspective on updating drug memories through the integration of memory editing and brain stimulation

**DOI:** 10.3389/fpsyt.2023.1161879

**Published:** 2023-04-14

**Authors:** Xavier Noël

**Affiliations:** ^1^Laboratoire de Psychologie Médicale et d’Addictologie, Faculty of Medicine, Université Libre de Bruxelles (ULB), Brussels, Belgium; ^2^Neuroscience Institute (UNI), Université Libre de Bruxelles (ULB), Brussels, Belgium

**Keywords:** addiction, memory reconsolidation, non-invasive brain stimulation, memory editing, substance use disorders

## Abstract

Addiction is a persistent, recurring condition characterized by repeated relapses despite the desire to control drug use or maintain sobriety. The attainment of abstinence is hindered by persistent maladaptive drug-associated memories, which drive drug-seeking and use behavior. This article examines the preliminary evidence supporting the combination of non-invasive brain stimulation (NIBS) techniques and memory editing (or reconsolidation) interventions as add-on forms of treatment for individuals with substance-related disorders (SUD). Studies have shown that NIBS can modestly reduce drug use and craving through improved cognitive control or other undetermined reasons. Memory reconsolidation, a process by which a previously consolidated memory trace can be made labile again, can potentially erase or significantly weaken SUD memories underpinning craving and the propensity for relapse. This approach conveys enthusiasm while also emphasizing the importance of managing boundary conditions and null results for interventions found on fear memory reconsolidation. Recent studies, which align with the state-dependency and activity-selectivity hypotheses, have shown that the combination of NIBS and behavioral interventions holds promise for treating SUD by reducing self-reported and physiological aspects of craving. Effective long-term outcomes for this procedure require better identification of critical memories, a deeper understanding of the brain mechanisms underlying SUD and memory reconsolidation and overcoming any boundary conditions of destabilized memories. This will enable the procedure to be personalized to the unique needs of individual patients.

## Introduction

The chronic and recurring nature of addiction creates a significant problem for healthcare providers and those affected, as the potential for relapse remains even after the acute withdrawal symptoms have passed (1, 2). This is due, in part, to the persistence of maladaptive memories associated with drug cravings and seeking behaviors (3–6).

Although there have been major strides in recent years in understanding the brain mechanisms of addiction through research in both animals and humans (7, 8), the clinical effectiveness of brain-targeted interventions in humans remains limited (2, 9–11). Wider research aimed at a comprehensive understanding of addictive behaviors from both neural and learning perspectives can investigate the brain’s function, mental content, and contextual factors involved in addiction onset and relapse. This effort would bring together three key perspectives: altering addiction-linked memory content, exploring brain stimulation’s role in memory updates, and utilizing neurocognitive insights into an addiction to inform memory-based neurocognitive interventions. The current article showcases this approach by examining interventions that combine memory editing and non-invasive brain stimulation (NIBS) in treating patients with substance-use disorders (SUD).

In the following section, I will discuss the present status of memory editing interventions employed in the context of SUD. By reactivating addiction-related memories using pharmacological methods, such as the application of the β-adrenergic receptor (βAR) antagonist propranolol—which works by mitigating the effects of stress hormones like adrenaline on beta receptors, subsequently reducing heart rate, blood pressure, and overall cardiac workload—or through behavioral interventions like extinction and counterconditioning, cravings and drug consumption can be diminished. I will also explore potential reasons why memory reconsolidation interventions did not yield the anticipated results, particularly in the context of fear responses, emphasizing the mechanisms and boundary conditions of memory reconsolidation that warrant consideration for SUD.

The project of utilizing behavioral interventions (e.g., memory reconsolidation) and NIBS for treating SUD will be further developed. The concept of “state-dependency” (12), which suggests that the effectiveness of NIBS is closely related to the activity levels of the specific brain region or network being targeted, is of great importance for this discussion. Research into the effects of NIBS on memory reconsolidation is emerging in the context of fear and post-traumatic stress disorder memories (13, 14), but it remains uncertain if this combined approach positively influences SUD treatment. An additional section will examine the existing literature on the combined strategy of applying NIBS to brain circuits involved in SUD alongside memory reconsolidation interventions, with the aim of determining whether this dual intervention can effectively reduce cue-induced craving and drug consumption. The review complements previous theoretical proposals (15, 16) by discussing recent data on this topic.

### Neurocognitive models of addictive behaviors

The intention of this section is not to present an exhaustive examination of neurocognitive models of addiction in animals and humans. These types of evaluations can be found in other reputable sources (7, 8, 17–20). Rather, its emphasis is on pinpointing particular attributes of these models that may lend support to the intervention strategy presently being expounded.

Dual-process theories of addiction suggest that addictive behaviors arise from both an impulsive neural system, which is heavily influenced by the amygdala and striatum and leads to the reinforcement of automatic and spontaneous memory patterns, and a reflective neural system, which is primarily controlled by the prefrontal cortex and is responsible for decision-making and deliberate, self-control (21, 22). An advanced version of this theory emphasizes the role of the insula serving as a hub of the interoceptive awareness system and interplaying neurocognitive systems involved in drug addiction (23, 24). The result arises from the described imbalanced interaction among reflexive, reflective, and internal perception processes. This disparity results in significant cognitive prejudices, including an increased fixation on addiction-related cues, a predilection for instant gratification over delayed rewards, and decreased self-control in addiction-related contexts. The gradual sensitization of the brain’s reward system to drugs and addictive behaviors leads to an enhanced attribution of incentive salience, or “wanting,” to the substance. This heightened desire results in intense urges that ultimately drive compulsive behaviors (19).

These theories, along with others (e.g., (25)), suggest that through practices over a long time, unconditioned and conditioned drug-related cues have developed strong motivational properties towards drug use. Specific stress factors play a role in amplifying the motivation to engage in drug use (26, 27).

To sum up, most theories propose that instances of loss of control transpire as a result of the need to regulate hyperactive conditioned responses. This is influenced by elevated salience attribution, heightened cravings, and impaired inhibitory responses. These phenomena aid in our comprehension of why entrenched, dysfunctional memories that foster ongoing drug-seeking conduct and demonstrate resistance to extinction are hallmark features of addiction.

### Theory-based interventions in addiction

In a clinical setting, it is desirable to implement interventions aimed at modifying the persistent maladaptive memories associated with drug addiction, which are the source of cravings, spontaneous behaviors, and weakened engagement of self-control.

While NIBS methods have been utilized as an adjunctive treatment for drug addiction, their clinical effectiveness is limited and temporary (10, 11, 28, 29). The primary goal is to reduce cravings triggered by cues, which is widely recognized as a major contributor to drug use and relapse (30–32).

NIBS can decrease addiction-related cravings and negative emotions by activating prefrontal control over automatic responses generated by the insular and amygdala-striatal networks and some crucial brain systems involved in addiction (18). To enhance the effectiveness of NIBS in clinical settings, experts recommend taking into account factors such as session timing, sample size, voltage level, personalized, targeted brain regions, comorbidity and psychological factors to guide the current state of the science and future direction (33).

A resurgent approach is extinction techniques, which involve exposing an individual repeatedly to a stimulus or memory with no accompanying punishment or reward, resulting in the understanding that the stimulus holds no significance (34). The aim is to directly alter the memory structure of individuals with SUD to weaken the impulses and internal responses that drive increased motivation to use drugs (35). However, traditional extinction methods do not erase the original learning but instead create new learning with inhibitory properties associated with the conditioned stimulus, which depends on context (36). This inhibition process highly depends on how the knowledge was acquired and the context in which it occurs. This can result in the spontaneous return of the original conditioned response following traditional extinction protocols. To address these limitations, a procedure has been developed to enhance the process of extinction and prevent the return of the original response, such as using memory reconsolidation as a technique of memory editing (34, 37, 38).

### The theory of memory reconsolidation

The research on reconsolidation was reignited after a 30-year gap, thanks to studies by Nader et al. (39), who reported the disruption of Pavlovian fear memories by administering anisomycin, an antibiotic that has been found to inhibit protein synthesis in cells, following retrieval. This research provided two key insights: firstly, that consolidated memories could be “erased” after retrieval, and secondly, that the underlying mechanism of this “reconsolidation” process mirrored the original consolidation in its need for protein synthesis.

The core concept of memory reconsolidation posits that when a consolidated memory is reactivated, it temporarily enters a fragile state for minutes to hours before re-stabilizing or reconsolidating. Significantly, both animal and human research have shown that the retrieval cue initiates a finite period of plasticity (believed to last no more than 6 h after reactivation) (39–41). Within this reconsolidation window, the memory is susceptible to behavioral interventions (such as extinction, counterconditioning, or interference) (37), allowing it to be updated or altered to remain pertinent in view of new information (39, 42). Once reconsolidated, the memory returns to a stable state and resists interference. Initially designed to eliminate maladaptive memories associated with negative (e.g., traumatic) events, which yielded significant but mixed outcomes (43–45), the modification of addiction-related memories have recently been explored and demonstrated promising results (44).

Importantly, in memory reconsolidation theory, prediction error plays a significant role in determining whether a memory will undergo reconsolidation or not (46, 47). Prediction error refers to the discrepancy between an individual’s expectation and the actual outcome of a situation or event. When a memory is reactivated, and the outcome differs from the original expectation, a prediction error occurs. In the context of reconsolidation, prediction error is thought to be a crucial factor that destabilizes the reactivated memory, allowing it to become malleable and open to modification (48). This destabilization is necessary for the memory to undergo reconsolidation, a process in which the memory trace is updated and strengthened with new information before being stored again. In other words, prediction error creates a window of opportunity for the memory to be altered, which can be leveraged in therapeutic interventions.

Therefore, the contribution of prediction error in memory reconsolidation theory is to signal that the current memory representation is not accurate, necessitating an update or modification to reflect the new information or experience better. This allows the memory to be more adaptive and relevant to the individual’s current context.

### Memory reconsolidation interventions in SUD

Memory reconsolidation intervention has effectively reduced drug-seeking behaviors in animals and humans (37, 43, 44, 49–54). Retrieving memories associated with drug use 10 min prior to extinction sessions reduced cue-induced heroin cravings for up to 180 days in patients undergoing detoxification for heroin addiction (55). Other studies showed that pre-retrieval propranolol administration blocked the re-stabilization of smoking-related cues. Administering the noradrenergic beta-receptor blocker propranolol immediately after a retrieval session of drug-related cue exposure (e.g., reading a personalized script) in polysubstance abusers (56) and cocaine-dependent individuals (57) decreases both subjective craving and psychophysiological arousal (i.e., heart rate and blood pressure) in response to these specific cues. A study by Lonergan (56) found that administering propranolol before drug use reduced craving in treatment-seeking adults with SUD but had no effect in the placebo group. In nicotine in smokers, administering propranolol before retrieving memories of an unconditioned stimulus reduced subsequent cravings triggered by previously associated cues (58). In abstinent heroin addicts, propranolol given before the reactivation of a word list impaired the reconsolidation of heroin-related words (59). Despite this, the reduction in cravings is not sustained (57, 60), and it did not affect the likelihood of relapse in nicotine dependence (61). A recent systematic review and meta-analysis revealed that impairing reconsolidation with propranolol led to the alleviation of psychiatric symptoms and decreased cue-induced reactivity in clinical populations, including those with addiction (44).

Other retrieval procedures that manipulated outcome expectancies to elicit prediction errors have been conducted with heavy alcohol drinkers (62, 63). Preventing individuals from drinking beer unexpectedly (omission prediction error) significantly decreased verbal fluency for positive alcohol-related words, suggesting a potential restructuring of memory (63).

The investigation into the neural underpinnings of propranolol-induced disruptions in reconsolidation is ongoing, with some significant findings already emerging. Generally, the interruption of fear memory reconsolidation primarily impacts the activation of the prefrontal cortex, amygdala, and hippocampus (44, 64). In studies of smokers, nicotine-related memories showed heightened connectivity between the hippocampus and striatum, which positively correlated with reduced cravings following reconsolidation manipulation in the propranolol group (65). This increased hippocampal activation suggests that propranolol interferes with memory reconsolidation and diminishes the rewarding aspects of smoking-related cues by modulating the activation of memory-associated brain areas. Nonetheless, there have been conflicting findings, such as the report that a single dose of propranolol does not impact physiological or emotional responses to smoking cues (60).

The clinical outcomes of the study, which focused on modulating reconsolidation and extinction to control drug reward memory, are promising. However, there are potential limitations that will be discussed in the subsequent section.

### Mechanisms and boundary conditions of drug memory reconsolidation

Given the inconclusive outcomes of fear reconsolidation interventions (45), using memory editing in a clinical setting for treating SUD poses numerous challenges and requires further examination.

In SUD, various forms of memory, such as habitual actions, episodic details, defensive responses, and subjective feelings like withdrawal, craving, and stress, are all involved. Each of these forms of memory involves different neural systems for storage and expression (17). Whether editing one form of memory alters other forms for the same event is unknown (43). Additionally, reactivating a memory requires specific conditions for retrieval. The ideal context, such as the timing or duration of retrieval, has yet to be determined and can vary depending on the age or strength of the memory.

Furthermore, the impact of individual differences on the effectiveness of retrieval-extinction may be significant. Indeed, many individuals with SUD encounter subjective limitations when emotions are activated (66). Although speculative, we anticipate that clinical interventions promoting the representation of emotion (67) are crucial to fully benefiting from a reconsolidation procedure.

Incorporating a mismatch between what is expected and what occurs during the procedure, known as prediction errors, seems to destabilize memories and may be beneficial in memory editing interventions (47, 68). However, individuals with SUD often have a disrupted capacity for generating prediction errors, which might be a limiting factor to editing memory satisfactorily (69). Spontaneous responses to the incentive value of cues, such as attentional biases towards addiction-related stimuli or avoidance action tendencies, can also impact the efficacy of memory reconsolidation. For example, recently detoxified alcohol-dependent individuals have been found to have avoidance action tendencies (70), which may make it more difficult to elicit a strong emotional response to alcohol-related cues. It may also be challenging to increase craving for alcohol through cue exposure in hospitalized patients, possibly due to limitations in creating realistic positive expectations associated with alcohol use (2) due to a low perceived drug use opportunity affecting responses to the presentation of drug cues (71). The data indicate that drug use is a goal-directed process that includes positive expectations and discrepancies between goals and situations (72, 73). As a result, it is likely to be beneficial to activate pertinent memories in real-world scenarios where drug use is acquired.

Additionally, stress may impact the reconsolidation of extinction memories (74). Whether induced behaviorally or through corticosterone administration, stress can negatively affect extinction retrieval and may even promote relapse (75). It is essential to consider these sources of variability to understand individual differences in response to reconsolidation interventions and when trying to generate craving through retrieval of a stressful personal experience (76). The next section will examine the possible effects of NIBS on enhancing memory editing.

### Potential benefits of combining NIBS and memory interventions for SUD: Initial findings

Initial findings on the potential benefits of combining NIBS with memory reconsolidation interventions for SUD.

While further investigation is required to determine the best protocols, fine-tune stimulation parameters, and pinpoint the ideal patient group for using NIBS interventions to address addictive behaviors (33), one key reason for the inconsistent and limited outcomes documented in the literature may also stem from the necessity of combining NIBS with additional behavioral interventions (9, 10, 77).

The “activity-selectivity hypothesis” provides an explanation for the potential benefits of combining NIBS with behavioral interventions (78). This theory suggests that when NIBS is applied to an already active brain region, the neuroplastic effects are stronger and more enduring. This idea is similar to the principle of “state-dependency” (12), which posits that the effectiveness of NIBS depends on the underlying state of the stimulated region or network. This principle has led to re-evaluating NIBS as an interaction between external stimuli and the brain’s internal state. In this review, several non-invasive brain stimulation (NIBS) techniques are explored and evaluated in the context of memory reconsolidation interventions in SUD. Please refer to Table 1 for a brief description of each technique.

**Table 1:** Most common non-invasive brain stimulation techniques used in psychopathology.

Technique	Description
Transcranial magnetic stimulation (TMS)	Uses rapidly changing magnetic fields to induce electrical currents in targeted brain regions.
Continuous theta-burst stimulation (cTBS)	A form of repetitive TMS that applies a pattern of high-frequency magnetic pulses to modulate excitability.
Transcranial direct current stimulation (tDCS)	Applies low-intensity, direct current to the scalp, enhancing or suppressing neuronal activity.
Transcranial alternating current stimulation (tACS)	Applies oscillating electrical currents to modulate brain activity at specific frequencies.

The “activity-selectivity hypothesis” is supported by research showing that tDCS-induced plasticity is task-dependent (79–82). Leveraging the capacity of NIBS to disrupt activity in specific brain areas selectively, these techniques have been employed to influence cerebral activity during consolidation and reconsolidation processes (83), with the overarching aim of modulating these processes. For example, a study using an event-related potential paradigm found that tDCS can modulate cortical plasticity in the auditory cortex activity-dependently (84). Positive cumulative or synergistic clinical effects when combining NIBS with behavioral interventions (i.e., specific-cue response inhibition training) have been found in individuals with a severe alcohol-use disorder (2). Other studies that used the dual approach in alcohol-dependent patients found no evidence to support the idea that tDCS specifically enhances cognitive bias modification (85, 86). A significant limitation of combining NIBS with repetitive behavioral interventions (i.e., training sessions) is the risk of participant disengagement due to fatigue or boredom. A recent study even reported iatrogenic effects, such as worse cognitive performance and clinical outcomes, when TMS sessions were combined with working memory training interventions in individuals addicted to nicotine (87). Thus, combining interventions such as NIBS with psychotherapies (9, 77) or memory reconsolidation (15, 88–90) has been proposed as a more feasible and ecologically valid approach in different contexts, such as fear memory and drug cravings. Now, let us examine the association between NIBS and memory reconsolidation interventions.

A recent study demonstrated that noninvasive stimulation of the prefrontal cortex after memory reactivation could disrupt physiological responses to learned fear and supports the critical role of the dorsolateral prefrontal cortex (dlPFC) in the neural network that governs the reconsolidation of fear memories in humans (88). It’s worth mentioning that enhanced extinction memory recall can also be achieved by using NIBS on the dlPFC during extinction learning (14). A study provided evidence supporting the efficacy of the dual approach in a clinical population. The study showed that high-frequency rTMS applied over the ventromedial PFC can improve exposure therapy response for acrophobia symptoms (91). In another recent study, continuous theta-burst stimulation (cTBS) applied over the right dlPFC after CS exposure effectively reduces fear response and prevents its return (89). The fear response was reduced when cTBS was delivered only during the reconsolidation window and was effective for both recent and remote fear memories. The impact of cTBS on reconsolidation depended on the delay, suggesting that cTBS has a disruptive effect on fear memory reconsolidation. A recent study surprisingly showed that repeated stimulation of the medial prefrontal cortex using deep transcranial magnetic stimulation might negatively affect the extinction of trauma memory for unknown reasons (13). Indeed, participants that underwent repeated ultra-brief exposure sessions combined with fake stimulation showed significantly greater improvement compared to those who received the same exposure and followed it up with active dTMS treatment. One potential reason could be that the brain stimulation device utilized in this study targeted the dorsal anterior cingulate cortex, which was not targeted in a previous feasibility study (92). This result underscores the importance of being cautious when selecting the brain circuits targeted by NIBS when combined with memory reconsolidation interventions.

A cue provocation procedure involving NIBS showed promising clinical outcomes for patients with inpatients with obsessive–compulsive disorder (OCD) who failed to benefit sufficiently from treatments with SRIs and cognitive and behavioral therapy (93). Results from OCD studies are of particular interest for our purpose since compulsivity has been considered a transdiagnostic factor also characterizing SUD (17). In this study, OCD patients were randomly assigned to receive either high-frequency (20 Hz) or sham deep transcranial magnetic stimulation (dTMS) targeting the medial prefrontal cortex and the anterior cingulate cortex following individualized symptom provocation provided at the beginning of each treatment session for 6 weeks (e.g., “*Please keep thinking about the dirty handle*”). The active dTMS treatment group showed the highest reduction in OCD scores, and this effect was maintained at the 1-month follow-up. Although promising, the impact of provocation was not controlled for; hence, the exact contribution of the exposure procedure is not fully known.

The combination of NIBS with reconsolidation interventions aiming to edit memories has been recently tested in SUD. A systematic review of NIBS studies in nicotine dependence reported six investigations using cue provocation (94). Some studies have reported positive outcomes, such as smoking reduction and reduced nicotine cravings, e.g., (95–98), while others have found negative results in these clinical aspects (99, 100). For instance, one study used TMS on the prefrontal and insular cortices with or without smoking cues to induce smoking cessation (97). Combining active TMS with exposure to smoking cues enhanced the reduction in cigarette consumption. This study highlighted the relevance of targeting the insula that integrates interoception states into conscious feelings (e.g., craving) (23, 24).

In another recent pilot study, intermittent theta-burst stimulation (iTBS) over the dlPFC or a sham condition was administered to individuals with methamphetamine use disorder after exposure to scenes depicting this drug use through virtual reality (90). The validation study shows that the virtual reality drug cue model in the online virtual world effectively differentiates high and low-craving meth abusers using heart rate variability (101). Active iTBS delivered 10 min after memory retrieval *via* virtual reality reduced self-reported craving scores and frontal q/b ratio (linked to attention bias to salient cues) in participants (90).

In conclusion, preliminary studies suggest that combining NIBS on the prefrontal regions and insula with re-exposure to past experiences may reduce drug cravings by disrupting outdated memories. Still, boundary conditions that need to be addressed have also been highlighted.

## Discussion

Investigations into the use of NIBS to weaken drug cravings by exposing participants to SUD-related cues and interfering during memory reconsolidation are just beginning, but they deserve attention given the lackluster efficacy of current clinical interventions (1). Many methodological and theoretical obstacles must be overcome for this dual therapy to be considered a viable clinical practice. However, interfering with the reconsolidation phase of memories in using NIBS involved in psychiatric conditions could potentially serve as an adjunct to evidence-based clinical interventions (16). For instance, continuous theta-burst stimulation over the right dlPFC delivered during the reconsolidation window of conditioned exposure of recent and remote fear memories decreases the fear response. It prevents recurrence (89). This approach has strong clinical prospects, especially when incorporated within a comprehensive clinical framework, like combining NIBS and psychotherapy in SUD (9, 77). I analysed the early research utilizing NIBS and memory reconsolidation in SUD, which showed a decrease in drug consumption and craving. Given the involvement of specific brain networks in SUD (e.g., in craving) (102), researchers have principally targeted the prefrontal regions and insula using NIBS. In the context of addiction, when selecting a brain region for applying NIBS, a primary criterion to consider is the technical accessibility of the involved area (33, 103). This is essential as the stimulation’s effectiveness relies on the ability to target and modulate neural activity in the relevant region precisely. The key factor is to determine which NIBS technique satisfies the technical accessibility requirements for the brain area implicated in SUD. By ensuring that the chosen region can be effectively targeted and modulated, the likelihood of successful intervention increases while the risk of adverse effects is minimized.

The combined approach is subject to the same limitations as each approach considered separately. Improving the optimal dosage and session number for targeting SUD-related brain regions through NIBS is still necessary (33), focusing on memory activation and reconsolidation.

The main challenge in the science of memory editing is understanding the various forms of memory (such as habits, episodic events, and subjective feelings) and how they relate to SUD in individuals (43). Indeed, the effectiveness of memory editing approaches can vary greatly depending on the learning process of a given individual (37). The process may be greatly aided when the outcome of the behavior (e.g., drinking) can be predicted and integrated into the learning process (71).

Intervention could alter expectations if situational factors and behaviors involve a representation of goals and expectations (72). A crucial aspect is that selecting a specific memory should be based on an individual’s goal-directed determinant of action, meaning a stimulus that elicits positive expectations and initiates a goal-directed cycle leading to drug use (73). Specifically, suppose participants are more likely to have rigid expectations of drinking’s positive outcomes during stressful, low-resource conditions. In that case, a memory reconsolidation intervention using stress to modify positive expectations and cravings is needed. When addictive behaviors stem from the connection between stimulus features and response representations without depending on the individual’s knowledge of outcomes (17), activating positive expectations and related craving may not be so determinant to make memory reconsolidation effective in reducing drug use. In either scenario, alterations in processes like attention biases for drug cues, drug-related implicit and explicit memory biases, and approach action tendencies towards drug stimuli are expected to drive changes in behavior in response to the dual intervention (5).

To effectively update original memories through memory reconsolidation interventions, it is necessary to reactivate emotional memories to some degree, although the precise amount has yet to be determined. This can be a barrier for individuals with difficulty identifying and expressing their emotions, as alexithymic tendencies can negatively impact the success of psychotherapy (66).

Other factors, such as memory age, the intensity, duration, or the number of presentations of the unconditioned and conditioned stimulus, and the optimal degree of mismatch between predicted and observed events should be considered in future research using a dual approach. Indeed, the combined system faces significant challenges like those encountered in memory update trials using the memory reconsolidation procedure.

At last, further research is needed to elucidate the role of brain networks, including the dlPFC and insula, in modifying these types of memories and how different NIBS techniques may aid in reconsolidating appetite memories (88, 89). While further research is needed, using a combination of these approaches has the potential to change outdated memories and decrease compulsive behaviors.

## Author contributions

XN substantially contributes to the conception or design of the work, to the writing process. He provided approval for publication of the content and agrees to be accountable for all aspects of the work in ensuring that questions related to the accuracy or integrity of any part of the work are appropriately investigated and resolved.

## Conflict of interest

XN is a Research Associate at the F.R.S./FNRS.

## Publisher’s note

All claims expressed in this article are solely those of the authors and do not necessarily represent those of their affiliated organizations, or those of the publisher, the editors and the reviewers. Any product that may be evaluated in this article, or claim that may be made by its manufacturer, is not guaranteed or endorsed by the publisher.
